# Reference growth curves for normal appendiceal diameter in childhood

**DOI:** 10.1038/s41598-020-69216-3

**Published:** 2020-07-22

**Authors:** Atsuhisa Fukuta, Toshihiko Kakiuchi, Eiji Sadashima, Takayuki Inoue, Katsumi Muramori

**Affiliations:** 1 Department of Pediatric Surgery, Saga-Ken Medical Center Koseikan, 400 Nakabaru, Kase-Machi, Saga, 840-8571 Japan; 2 Department of Pediatric Surgery, Reproductive and Developmental Medicine, Graduate School of Medical Sciences, Kyushu University, 3-1-1, Maidashi, Higashi-ku, Fukuoka, 812-8582 Japan; 3 Department of Pediatrics, Faculty of Medicine, Saga University, 5-1-1 Nabeshima, Saga, 849-8501 Japan; 4 Life Science Research Institute, Saga-Ken Medical Center Koseikan, 400 Nakabaru, Kase-Machi, Saga, 840-8571 Japan

**Keywords:** Anatomy, Gastroenterology, Medical research

## Abstract

The aim of this study was to investigate the relationship between the appendiceal diameter (AD) and age, sex, height, and body weight in children and to verify how does the normal AD grows in childhood. We evaluated the AD of patients younger than 16 years of age who underwent laparoscopic surgery at our hospital. We statistically examined the relationship between the AD and the age, sex, height, and weight. A final cohort of 188 patients participated in the study. The median AD for the sample population was 5 mm (range, 3.2–8.1). There was no significant difference in the AD between males and females in the multivariate analysis (*P* = 0.500). There was a positive correlation between the age and the AD (*R* = 0.396, *P* < 0.001). The AD had a significant positive correlation with the height and weight (*P* < 0.001, *P* < 0.001, respectively). The reference curve with regard to the AD can be useful in clinical situations, although it should be kept in mind that the range of individual differences in AD is large, and the growth degree by age is not uniform during childhood.

## Introduction

Acute appendicitis is one of the most common reasons for acute abdominal surgery in children^1,2^. There is a 7–9% lifetime risk of developing appendicitis^3,4^.

Recently, the early and accurate diagnosis of acute appendicitis has improved because of developments in diagnostic imaging technology. Ultrasonography is the first and most frequently used diagnostic method for acute appendicitis^5,6^. Computed tomography (CT) is used to confirm the diagnosis of appendicitis by ultrasound^7^. Some scholars have stated recently that magnetic resonance imaging (MRI) can be used for the evaluation of appendicitis in children^8,9^. However, the preoperative diagnosis of childhood appendicitis remains difficult for surgeons, because theirs presentation of appendicitis is nonspecific. Unfortunately, the rate of negative appendectomy (i.e. non-therapeutic surgical intervention) has been reported to range from 3 to 13%^10–14^.

The appendiceal diameter (AD) is an important parameter for the diagnosis of appendicitis. Evaluating the diameter of the normal appendix is important for improving the accuracy of the diagnosis. In some studies, the cut-off value for the AD has been calculated from the examination of images^15,16^. Searle et al. reported cut-off values for the AD from excised appendices^17^. However, studies directly evaluating the AD of a normal appendix in children have not been carried out. It is important to compare the most accurate direct measurement of the AD with the findings from previous reports that used imaging examinations or excised specimens for measurement.

The aim of this study was to investigate the relationship between the AD and age, sex, height, and body weight in children and to verify how does the normal AD grows in childhood.

## Methods

### Data collection

We evaluated the AD in children younger than 16 years of age who underwent laparoscopic surgery at our hospital between November 1, 2015, and August 31, 2017. Patients with appendicitis, abdominal inflammation, a history of appendicitis, or who had abdominal surgery were excluded. Only in cases where patient consent was given in advance did we measure the AD as part of the intraperitoneal observation at the time of laparoscopic surgery.

For AD measurements, we used a soft plastic surgical ruler with a width of 3 mm and length of 10 mm. This ruler includes a nylon thread to improve the safety of measurements and help prevent loss. We inserted this ruler via a 5- or 12-mm umbilical port and measured the AD at 2 sites at least (tip, middle, and root), verified the measurement results with video and photos after surgery, and used the mean value of the measurement results. If the measurement of the appendix was difficult because of adhesion or position, we discontinued measurement and did not add forceps or extra ports for measurement. The ages of children were recorded in years.

### Statistical analyses

We analyzed the relationship between the AD and the age, sex, height, and body weight. The relationship between the sex and continuous variables was evaluated using Wilcoxon’s rank-sum test. The association between the AD and the age was examined by Spearman’s rank correlation test.

We estimated the AD percentile curves for children by age, height and weight using the LMS and GAMLSS methods with the R package 'gamlss'. The 5th, 25th, 50th, 75th and 95th percentile values were calculated by age, height and weight for AD.

Statistical analyses were performed using the software programs SAS v9.4 (SAS Institute, Cary, NC, USA) and R v3.4.4 (R Foundation, Vienna, Austria). A correlation coefficient of > 0.3 was considered to be statistically correlated. A *P* value of < 0.05 was considered to be statistically significant.

### Ethical approval of the study protocol

Written informed consent was obtained from all participants and their parents or guardians. This study was performed according to the Ethical Guidelines for Clinical Research published by the Ministry of Health, Labour and Welfare of Japan on July 30, 2003 (revised in 2008) and complied with the Declaration of Helsinki (revised in 2008). This study was approved by the ethics committee for clinical research of Saga-Ken Medical Center Koseikan (17-07-02-03) prior to commencement of the study.

## Results

A total of 350 out of 556 patients were excluded from this study due to reasons such as different surgical sites, surgery other than laparoscopic surgery, and inflammation of the abdomen including appendicitis. Eighteen patients were excluded because they did not give consent for measurement of the AD or in whom the AD was difficult to measure. A final cohort of 188 patients participated in the study.

In this final cohort, there were 181 cases of inguinal hernia and hydrocele, 2 cases of urachal remnants, 2 cases of cryptorchidism, 1 case of varicocele, and 2 other laparoscopic surgeries. The age of patients who participated ranged from 0 to 15 years. In addition, 118 (62.8%) patients were male and 70 (37.2%) were female.

The median AD for the whole cohort was 5.0 mm (range, 3.2–8.1 mm). The median AD for males was 5.0 mm (range, 3.5–8.1 mm), and that for females was 5.1 mm (range, 3.2–7.3 mm). There was no significant difference in the AD between males and females in the multivariate analysis (*P* = 0.500).

**Figure 1:**
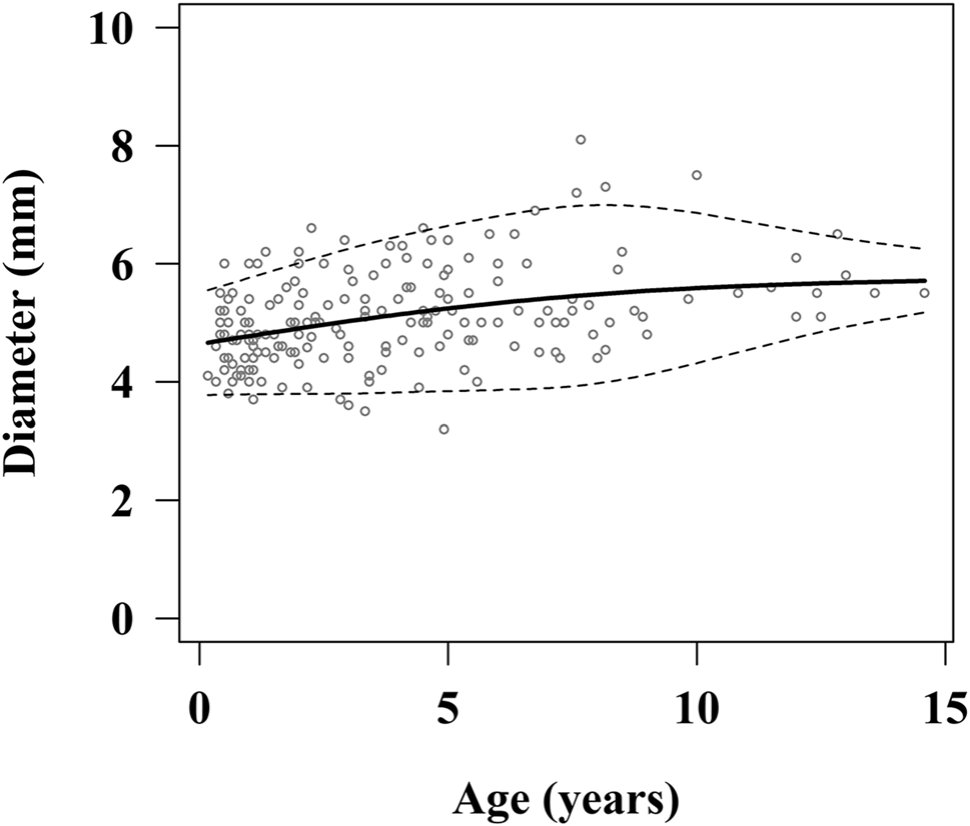
The reference curve between the appendiceal diameter and age. *Dotted line shows the 5th and 95th percentile values.

There was a positive correlation between the age and AD (*R* = 0.396, *P* < 0.001). Figure 1 shows the reference curve and the 5th and 95th percentile values with regard to the AD and age. There was also a significant positive correlation between the age and AD in the multivariate analysis adjusted for sex (*P* < 0.001). Table 1 shows the results of the LMS method analysis of each age with regard to the AD.

**Table 1:** Results of a LMS method analysis with regard to the AD and age.

Age (year)	5th	25th	50th (median)	75th	95th
0.5	3.78	4.33	4.71	5.09	5.63
1	3.79	4.37	4.77	5.18	5.76
1.5	3.79	4.41	4.84	5.27	5.89
2	3.79	4.45	4.90	5.36	6.01
2.5	3.80	4.49	4.97	5.45	6.14
3	3.80	4.52	5.03	5.53	6.25
3.5	3.81	4.56	5.08	5.61	6.36
4	3.82	4.60	5.14	5.68	6.46
4.5	3.83	4.63	5.19	5.75	6.55
5	3.84	4.67	5.24	5.82	6.64
5.5	3.85	4.70	5.29	5.88	6.73
6	3.86	4.73	5.33	5.94	6.81
6.5	3.87	4.76	5.38	5.99	6.88
7	3.89	4.79	5.41	6.04	6.93
7.5	3.92	4.82	5.45	6.07	6.97
8	3.97	4.86	5.48	6.10	6.99
8.5	4.04	4.91	5.51	6.12	6.99
9	4.12	4.96	5.54	6.12	6.96
9.5	4.21	5.01	5.56	6.12	6.92
10	4.31	5.07	5.59	6.11	6.86
10.5	4.42	5.12	5.61	6.09	6.79
11	4.53	5.18	5.62	6.07	6.72
11.5	4.64	5.23	5.64	6.05	6.64
12	4.75	5.28	5.65	6.03	6.56
12.5	4.84	5.33	5.67	6.01	6.49
13	4.93	5.37	5.68	5.99	6.43
13.5	5.02	5.41	5.69	5.97	6.36
14	5.09	5.45	5.70	5.95	6.31
14.5	5.16	5.49	5.71	5.93	6.26

*AD* appendiceal diameter.

**Figure 2:**
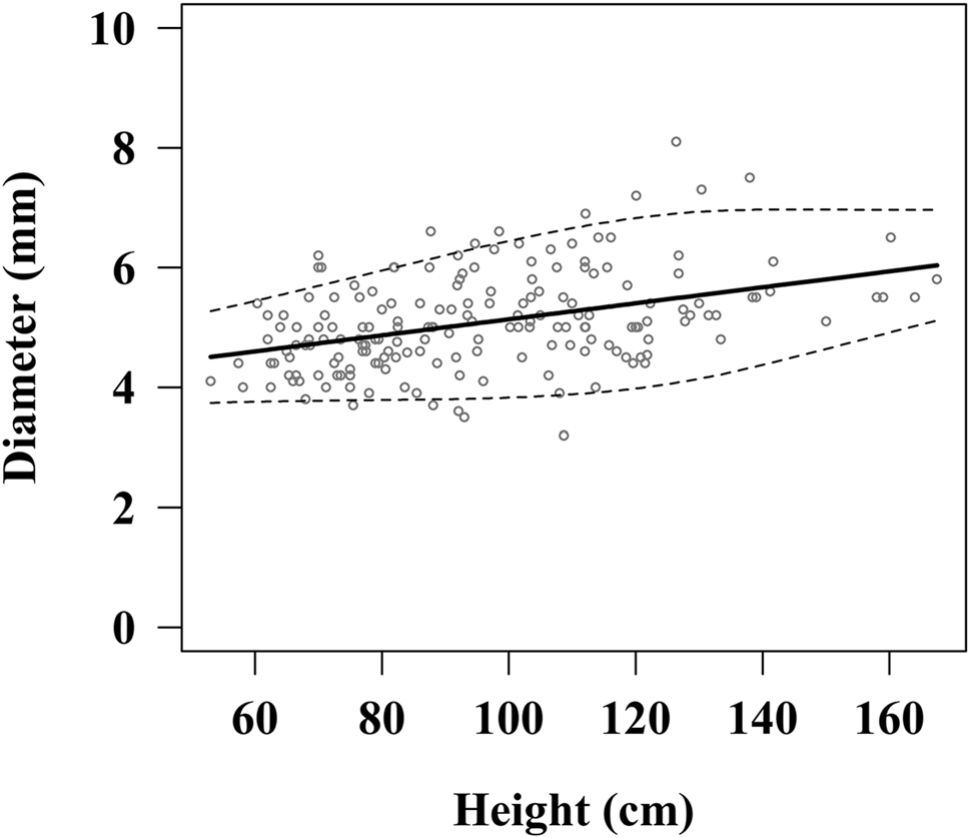
The reference curve between the appendiceal diameter and height. *Dotted line shows the 5th and 95th percentile values.

There was a significant positive correlation between the height and the AD for all ages (*R* = 0.417, *P* < 0.001), and Fig. 2 shows the reference curve and the 5th and 95th percentile values with regard to the AD and height. Table 2 further presents a summary showing the median value and the percentile values of the AD per 10 cm in height; the AD increasing rate was almost uniform with each height.

**Table 2:** Results of a LMS method analysis with regard to the AD and height.

Height (cm)	5th	25th	50th (median)	75th	95th
60	3.76	4.26	4.60	4.95	5.44
70	3.78	4.34	4.73	5.13	5.69
80	3.79	4.43	4.87	5.31	5.95
90	3.80	4.51	5.00	5.49	6.20
100	3.83	4.60	5.14	5.67	6.44
110	3.88	4.70	5.27	5.84	6.66
120	3.98	4.82	5.40	5.99	6.83
130	4.14	4.97	5.54	6.11	6.93
140	4.37	5.14	5.67	6.20	6.97
150	4.64	5.33	5.80	6.28	6.97
160	4.91	5.52	5.94	6.36	6.96

*AD* appendiceal diameter.

**Figure 3:**
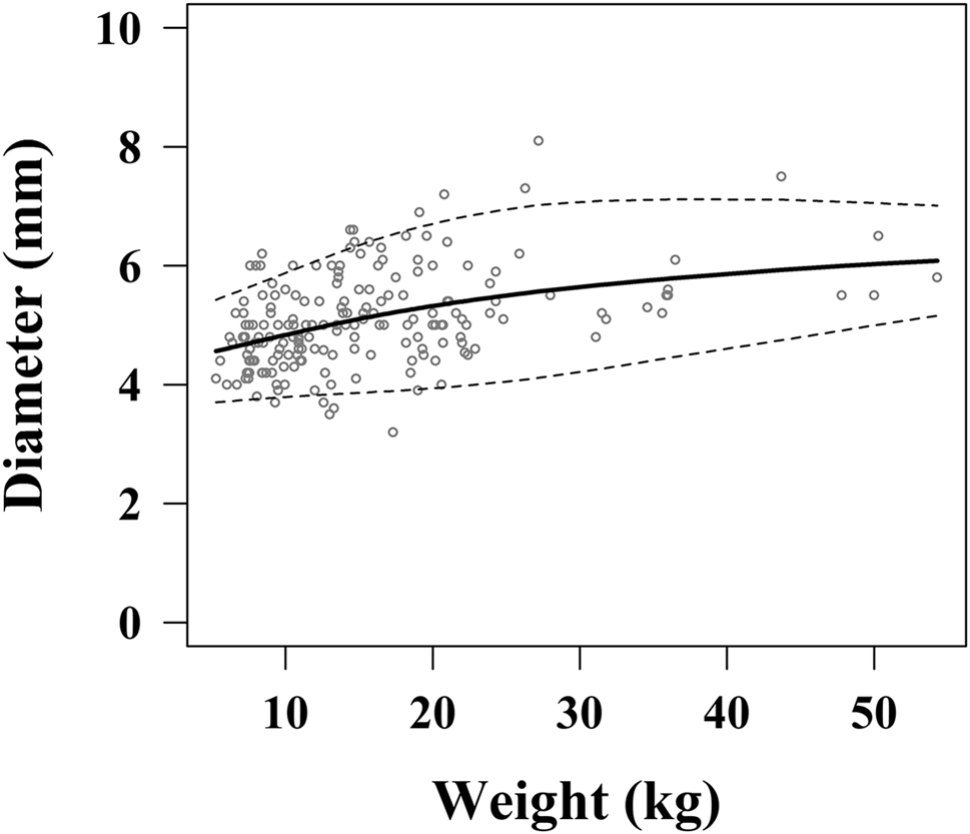
The reference curve between the appendiceal diameter and weight. *Dotted line shows the 5th and 95th percentile values.

There was a significant positive correlation between the weight and AD for all ages (R = 0.429, *P* < 0.001), and Fig. 3 shows the reference curve and the 5th and 95th percentile values with regard to the AD and weight. Table 3 shows the results of the LMS method analysis with regard to the AD and weight.

**Table 3:** Results of a LMS method analysis with regard to the AD and weight.

Weight (kg)	5th	25th	50th (median)	75th	95th
5	3.69	4.20	4.55	4.90	5.40
10	3.79	4.41	4.84	5.26	5.88
15	3.86	4.59	5.10	5.61	6.34
20	3.94	4.75	5.32	5.89	6.71
25	4.05	4.91	5.50	6.09	6.95
30	4.21	5.05	5.64	6.23	7.07
35	4.41	5.20	5.76	6.31	7.11
40	4.60	5.35	5.86	6.38	7.12
45	4.80	5.48	5.95	6.42	7.10
50	5.00	5.60	6.03	6.45	7.05
55	5.18	5.72	6.09	6.47	7.00

*AD* appendiceal diameter.

## Discussion

The AD is an important parameter for the diagnosis of acute appendicitis in children^16^. The evaluation of the diameter of the normal appendix is useful for improving the accuracy of the diagnosis. Several studies have reported on the evaluation of the AD using ultrasonography, CT, and MRI^8,16,18^. However, the assessment of normal appendix by images may be influenced by motion, the resolution of the image, or the accuracy of the equipment. One study has reported on the evaluation of AD from excised appendices^17^, but the assessment of AD using histopathology may be affected by the type of resection procedure and sample storage conditions. Although direct laparoscopic measurement can avoid such adverse effects and assess the diameter of the normal appendix, there have been no reports to date of such an approach. This is the first report on the direct measurement of the diameter of the normal appendix in children.

An AD > 6 mm is frequently used as the diagnostic cut-off for an abnormally enlarged or distended appendix^19,20^. In our study, the median AD in the whole cohort was 5 mm. This result does not contradict the cut-off value of 6 mm reported in previous papers. However, there were some individual differences in the AD in our study. Given the minimum AD of 3.2 mm and maximum of 8.1 mm, and seeing as 31 patients (16.4%) had an AD > 6 mm, individual variations were quite large in this study. Of the 31 cases, 1 (3.2%) was < 1 year old, 19 (61.3%) were between 1 and 6 years old, and 11 (35.5%) were > 6 years old.

Several scholars have reported that there is no correlation between the AD and age^18,21^, although some have reported the relevance of the AD^16,17^. Trout et al. examined the AD retrospectively using CT and found it to increase gradually until 6.5 years of age^16^. Searle et al. examined the AD retrospectively using histology data and noted that the AD increased until 3 years of age and that the appendix did not continue to grow throughout childhood^17^. In our study, there was a positive correlation between the age and AD, as increasing age slowed the AD growth. This result is similar to that reported by Searle et al.^17^, and this finding highlights the risk of using the same cut-off value in all age groups.

Regarding other findings from our study, the AD increased significantly with increasing body weight and height. In particular, the AD showed a constant rate of increase with increasing height. This result suggests that the height can be a useful index of AD.

This study has some limitations. First, we did not measure the AD in abnormal appendices. In order to obtain the accurate cut-off value of the AD for the diagnosis of acute appendicitis, it is necessary to evaluate not only the normal appendix but also the abnormal appendix. Next, nearly all of the samples had inguinal hernias/hydrocele. No previous reports have noted a relationship between appendicitis and inguinal hernias/hydrocele, except in cases of Amyand's hernia. There were no cases of Amyand's hernia in this study. For this reason, we consider the growth trend of AD in this study to be similar to that of "normal" AD. Third, all participants in this study were Japanese. Although the reference curves related to age may differ among populations, as different races have different growth curves, those related to weight and height may be useful in other populations. Finally, there was the bias of patients age. In other words, there was a high proportion of lower age groups. Acute appendicitis is not common in infants and younger children, but there is the risk of acute appendicitis in all ages including newborns. Furthermore, the rate of misdiagnosis rises as age decreases, and young children have a fivefold risk of complicated appendicitis^22,23^. That's why the AD of appendix diameters of younger age groups of this study is meaningful.

In conclusion, our study showed that AD growth is positively correlated with age, height, and weight. In particular, the AD showed a constant rate of increase with increasing height. The reference curve with regard to the AD can be useful in clinical situations, although it should be kept in mind that the range of individual differences in AD is large, and the growth degree by age is not uniform during childhood.
